# Salt Stress Re‐Routes Terpenoid and Flavonoid Metabolism in Peppermint ( *Mentha* × *Piperita* L.)

**DOI:** 10.1111/ppl.70694

**Published:** 2025-12-18

**Authors:** Francesco Caldo, Giuseppe Mannino, Massimo E. Maffei

**Affiliations:** ^1^ Department of Life Sciences and Systems Biology University of Turin Turin Italy

**Keywords:** flavonoid glycosides, menthofuran, menthol, metabolomics, photosynthesis, terpene gene expression

## Abstract

Salt stress is a major abiotic factor limiting crop productivity. This study investigated the physiological and biochemical responses of peppermint (*Mentha* × *piperita* L.) to increasing NaCl concentrations (25, 50, and 100 mM). Terpenoids and flavonoids were analysed by GC–MS and HPLC‐ESI‐MS/MS, respectively, alongside Real‐Time PCR for key biosynthetic genes (menthol biogenesis/Chalcone isomerase) and chlorophyll fluorescence measurements (OJIP, quantum yield, NPQ and PAM). Total terpenoid production decreased significantly (41% at 25 mM, 75% at 100 mM NaCl). Key compounds like menthone (365–71 g/kg), menthol (35–19 g/kg), and 1,8‐cineole (58–19 g/kg) were reduced, although pulegone and menthofuran initially increased at moderate salt levels (25 mM) before declining at 100 mM. Mechanistically, this suppression was due to a strategic metabolic bottleneck: upstream terpenoid biosynthetic genes (*DXS*, *GDPS*, *LS*, and *L3OH*) were upregulated, while terminal‐step genes (like menthone/menthol reductase) were progressively downregulated. This metabolic shift correlated strongly with severe photosynthetic impairment, evidenced by reduced quantum yield (0.82–0.61) and altered chlorophyll quenching (0.45–0.28). This impairment likely reduced the precursor supply for terpenoid synthesis. The plant also exhibited selective flavonoid adjustment. While total phenolic content (12.5–7.8 mg GAE g^−1^ FW) and regulatory gene expression were reduced, the biosynthesis of specific flavonoid conjugates increased (+1.5‐fold at high NaCl). These findings reveal that salt stress in peppermint triggers sophisticated and selective metabolic reallocation, severely impacting commercially valuable terpenoids and overall photosynthetic efficiency.

## 1 Introduction

Abiotic stress, particularly salinity, represents a major threat to global agriculture, significantly limiting crop productivity and plant survival in arid and semi‐arid regions (Trejo‐Tellez 2023). Salt stress disrupts critical plant functions, leading to reduced crop yield and quality, which has substantial economic consequences (Ahmad et al. 2023). Peppermint (*Mentha* × *piperita* L.) is a globally significant aromatic and medicinal plant, cultivated for its high commercial value derived from an essential oil rich in terpenoids, most notably menthol (Salehi et al. 2018; Zhao, Ren, et al. 2022; Hudz et al. 2023). Terpenoid biosynthesis is highly susceptible to environmental perturbation. While some studies indicate declines under both biotic and abiotic stress (Boncan et al. 2020), the response is highly dependent on stress type, intensity, and duration. Conversely, some reports suggest certain abiotic stresses, like UV radiation, can enhance essential oil content, implying complex and context‐dependent regulation of secondary metabolism (Maffei et al. 1999; Maffei and Scannerini 2000; Hazrati et al. 2025). Given the growing concerns over soil salinization, there is a critical need to understand how salt stress specifically affects this metabolism in economically important peppermint (Tedeschi et al. 2023).

In peppermint, monoterpenoid biosynthesis primarily occurs via the plastid‐localized 2‐C‐methyl‐d‐erythritol‐4‐phosphate (MEP) pathway (Perez‐Gil et al. 2024). Photosynthetic carbon fixation supplies much of the pyruvate and glyceraldehyde‐3‐phosphate (GAP) that initiate the MEP pathway (Lichtenthaler et al. 2006), leading to the universal C_5_ isoprenoid precursor, isopentenyl pyrophosphate (IPP). This direct link suggests that any reduction in photosynthetic efficiency, such as that caused by salinity, can directly limit the availability of carbon precursors for terpenoid production. In peppermint, key enzymes in terpenoid biosynthesis include isopentenyl pyrophosphate isomerase (IDI), geranyl diphosphate synthase (GDPS), (−)‐limonene synthase (LS), pulegone reductase (PR), and (−)‐menthone reductase (MR), while menthofuran synthase (MFS) diverts precursors to menthofuran (Johnson et al. 2017). Understanding the transcriptional regulation of genes coding for these enzymes is crucial for comprehending how plants control essential oil yield under environmental stress.

Previous studies have demonstrated that salt stress alters peppermint essential oil composition by typically decreasing menthone, menthol, and 1,8‐cineole, while sometimes reporting intermediate increases in compounds like pulegone and menthofuran (Aziz et al. 2010; Li et al. 2015; Zhao, Wei, et al. 2022; Kumar et al. 2023). However, a significant gap remains in our understanding of the underlying molecular and metabolic coordination that drives these changes. Plant stress responses are coordinated across multiple metabolic and physiological systems. Photosynthesis is the primary source of energy and carbon precursors (Hohmann‐Marriott and Blankenship 2011), not only for terpenoid biosynthesis (Melis 2013) but also for the production of flavonoids (Kołton et al. 2022), which act as crucial defensive and antioxidant molecules (Singh and Sharma 2015; Mathesius 2018). This integrated response often involves a metabolic trade‐off. For instance, under abiotic stress, peppermint shifts resources away from commercially desirable terpenoids toward protective compounds like kaempferol and quercetin glycosides (Dolzhenko et al. 2010). These flavonoids are potent antioxidants (Jan et al. 2022), and contribute to osmoregulation, helping maintain cellular water balance under saline conditions (Singh et al. 2022). The glycosylation of these compounds increases their solubility and stability, enhancing their effectiveness as protectants (Carrillo‐Martinez et al. 2024). Currently, a comprehensive model explaining the co‐regulation of photosynthesis, terpenoid metabolism, and selective flavonoid biosynthesis under salinity stress is limited.

This study aims to bridge that gap by investigating the physiological, biochemical, and molecular changes that occur in peppermint plants under increasing NaCl concentrations. We specifically focused on the plant's photosynthetic efficiency, the full profile of monoterpenoid production and associated gene expression, and the selective metabolic shifts in its phenolic and flavonoid profiles.

## 2 Materials and Methods

### 2.1 Plant Material and Growth Conditions

Young peppermint plants (*Mentha* × *piperita* L., Black Mitcham variety), approximately 2 months old, were collected in May from a field in Moretta (Piedmont, Italy). The rooted plants were then transferred to our laboratory, transplanted into 12 cm diameter pots with standard soil, and acclimated for a 2‐week period with tap water irrigation. Homogeneous cuttings were then obtained and placed in 250 mL Erlenmeyer flasks containing either a control solution (distilled water supplemented with half‐strength Murashige and Skoog (MS) medium; Sigma) or the control solution supplemented with increasing concentrations of sodium chloride (NaCl): 25, 50, and 100 mM. These concentrations were chosen based on previous studies of salinity stress in peppermint (Profico et al. 2025). Plants were maintained for 7 days under controlled environmental conditions: 25°C ± 2°C, 60%–70% relative humidity, and a 16/8 h light/dark photoperiod with a light intensity of approximately 150 μmol m^−2^ s^−1^. This short timeframe was selected to capture the early metabolic and physiological responses of peppermint to salt stress, before significant growth inhibition or tissue damage. After 7 days, the plants were harvested for analysis. The experimental design included three biological replicates (separate flasks) per condition, with three cuttings taken from each flask, resulting in a total of nine plant samples per treatment.

### 2.2 Terpenoid Extraction and Analysis

Approximately 5 g of leaves from both control and treated plants were flash‐frozen in liquid nitrogen and ground to a fine powder using a mortar and pestle. Terpenoids were extracted from the powdered tissue in screwcap tubes containing hexane with 0.2 mg/mL camphor as an internal standard. The tubes were vortexed for 30 s and then sonicated in a cold water bath for 15 min to ensure a complete extraction. The supernatant was collected after centrifugation and passed through anhydrous magnesium sulphate (MgSO_4_) to remove residual water. The solvent was then evaporated under a stream of N_2_ gas, and the concentrated extract was stored at −20°C until analysis.

Terpenoid identification was performed using gas chromatography–mass spectrometry (GC–MS; 5975 T, Agilent Technologies). Quantitative determination was carried out using gas chromatography with a flame ionization detector (GC‐FID; GC‐2010 Plus, Shimadzu) as previously described (Dolzhenko et al. 2010).

### 2.3 Photosynthetic Pigments and Total Phenols Quantification

Chlorophyll *a*, chlorophyll *b*, and total carotenoids were extracted and quantified using a UV1280 spectrophotometer (Shimadzu) following the protocol of Lichtenthaler and Wellburn (1983). The total phenolic content was determined spectrophotometrically using the Folin–Ciocalteu's reagent, which involves the reduction of phosphotungstic‐phosphomolybdic acid to blue pigments in an alkaline solution (Medina 2011).

### 2.4 Chlorophyll *a* Fluorescence Kinetics

Chlorophyll fluorescence parameters, including OJIP kinetics, quantum yield, non‐photochemical quenching (NPQ), and pulse amplitude modulated (PAM) fluorescence, were measured using an FP100 fluorometer (Photon Systems Instruments). Measurements were performed according to the manufacturer's instructions and the method of Strasser et al. (2000). For each plant, fully expanded young leaves were selected and dark‐adapted for 30 min prior to measurement to ensure that all Photosystem II (PSII) reaction centres were open. Fluorescence induction curves (OJIP transients) were recorded using a saturating light pulse, allowing the evaluation of the maximum quantum efficiency of PSII (F_v_/F_m_) and other parameters reflecting the activity of PSII. NPQ and effective quantum yield were evaluated under actinic light conditions to estimate the plant's photoprotective capacity and energy dispersion mechanisms under salt stress. Three biological replicates were measured for each treatment, sampling multiple leaves per plant to account for intra‐plant variability. Data were collected and analysed using FluoPen software (1.1.0.3, Photon Systems Instruments), with parameters calculated according to standard formulas reported in the literature (Strasser et al. 2000).

### 2.5 Flavonoid Characterization of by HPLC‐DAD‐ESI‐MS/MS

The identification and quantification of flavonoids in peppermint extracts were performed using high‐performance liquid chromatography (HPLC) coupled to a diode array detector (DAD) and tandem mass spectrometry (MS/MS). The system included an Agilent Technologies 1200 HPLC system coupled to a Bruker Daltonics MS/MS system with an electrospray ionization (ESI) source. Separation was performed with a Luna C18 column (4.6 × 150 mm, 5 μm particle size, Phenomenex). The chromatographic separation was carried out at 30°C with a flow rate of 0.1 mL/min using a binary gradient of water with 0.1% formic acid (solvent A) and acetonitrile (solvent B), as previously described (Mannino et al. 2023). Data acquisition was performed in multiple reaction monitoring (MRM) mode as previously reported (Parmagnani et al. 2022). Flavonoids were identified by matching retention times, UV spectra, and MS/MS fragmentation patterns with authentic standards and literature data. The limit of detection (LOD) and limit of quantification (LOQ) for each flavonoid were calculated according to the method described by Salem et al. (2020).

### 2.6 RNA Preparation, cDNA Synthesis, and qRT‐PCR Assays

Total RNA was isolated and purified from peppermint leaves using Peqlab PeqGOLD TriFast reagent (VWR Avantor). The concentration of extracted RNA was determined using a BioSpec‐nano spectrophotometer (Shimadzu). For cDNA synthesis, 500 ng of total RNA was reverse‐transcribed using qScript Ultra Supermix (Quantabio) following the manufacturer's instructions. Quantitative Real‐Time Polymerase Chain Reaction (qRT‐PCR) was performed on a QuantStudio 3 Real‐Time PCR System (Applied Biosystems). The 10 μL reaction mixture contained 5 μL of 2× Perfecta SYBR Green Fastmix qPCR Master Mix, 0.25 μL of cDNA, and 0.01 nmol of primers (Integrated DNA Technologies). Perfecta SYBR Green Fastmix included ROX as an internal loading standard. The relative transcript level of each gene was calculated using the *ΔΔ*C_t_ method, with the expression of the *UBQ* gene serving as the internal reference. Two Chalcone‐flavanone Isomerase (*CHI*) isoforms were also studied. Specifically, using the Arabidopsis sequences of the AT3G55120 and AT5G05270 genes, we performed a BLAST search against the peppermint EST database. The search identified the peppermint sequence MW173ED (AW255925) as a high‐homology match for AT3G55120, and ML485 (AW255462) as a high‐homology match for AT5G05270. Primers for qRT‐PCR were designed using the Primer3 software and are detailed in Table S1.

### 2.7 Statistical Analysis

Statistical analyses were performed on data from at least three biological replicates. Data are presented as the mean and standard deviation. The significance of differences between treatments was assessed using one‐way ANOVA followed by a post hoc Tukey test. Cluster analysis and principal component analysis (PCA) were conducted using Systat10. A heatmap of the data presented in Figure S2 was generated using Heatmapper (http://www.heatmapper.ca/) (Babicki et al. 2016) with Pearson clustering and the single linkage method.

## 3 Results

### 3.1 Salt Stress Reduced Terpenoid Production in Peppermint (*Mentha* × *Piperita*)

Exposure of peppermint cuttings to increasing concentrations of NaCl elicited a typical salt stress response. Plants grown under control conditions developed normal root systems and green leaves, whereas those exposed to increasing NaCl concentrations exhibited progressive stress symptoms, including curled and crispy leaves (Figure S1).

**FIGURE 1:**
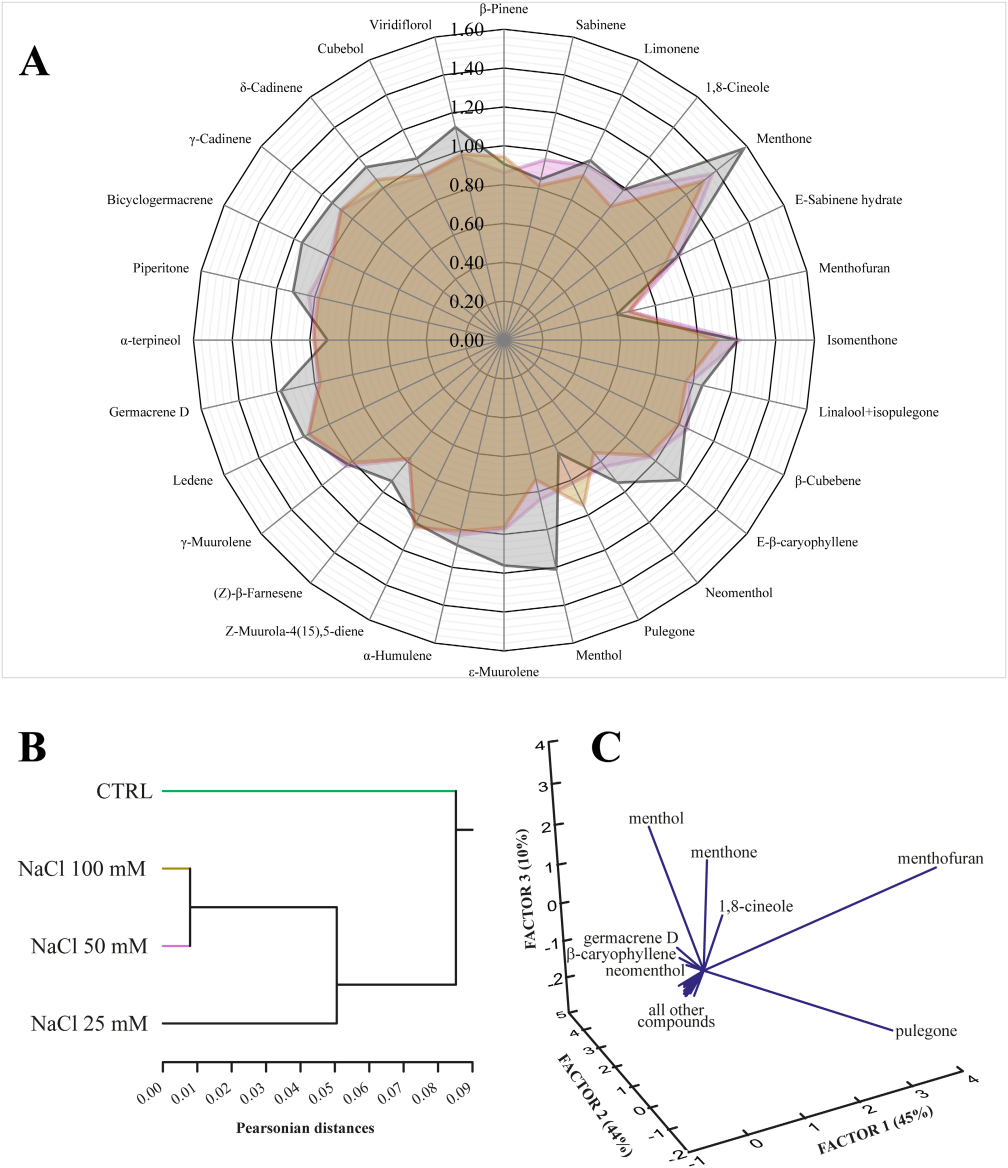
Peppermint ( *Mentha* × *Piperita* ) terpenoid response to increasing NaCl concentrations. (A) Radar plot showing the relative GC composition of terpenoid classes in plants exposed to different NaCl concentrations. (NaCl 25 mM, grey colour; NaCl 50 mM, pink colour; NaCl 100 mM, brown colour). Values represent log_2_ fold change of relative percentage (Rel%) relative to control. (B) Cluster analysis using Pearsonian distance with the single linkage method, depicting the relationship between terpenoid profiles under different NaCl concentrations. (C) Principal component analysis (PCA) of terpenoid responses, showing how individual compounds contribute to the overall variance in the terpenoid profile.

GC–MS analysis of the extracted volatile fraction revealed a significant reduction in total monoterpene and sesquiterpene content in NaCl‐treated plants compared to controls. Specifically, exposure to 25 mM NaCl resulted in a 41% reduction in total terpenoid production, which further decreased to 75% at 100 mM NaCl. Interestingly, the 50 mM NaCl treatment caused a 34% reduction, statistically comparable to the 25 mM treatment, although visual symptoms were more pronounced (Table 1; Figure S1). To further illustrate the compositional changes, a radar plot representing the GC composition data was generated (Figure 1A). Values were normalized as log_2_ fold change of relative percentage (Rel%) for the different experimental conditions relative to the control. This plot highlights that the reduction in specific terpenoid classes becomes more pronounced at higher NaCl concentrations, providing a clear overview of the shifts in terpenoid composition under salt stress.

**TABLE 1:** Monoterpenes and sesquiterpenes of peppermint (*Mentha × piperita*) control plants and plants exposed to increasing concentrations of NaCl.

Compound	Control		NaCl 25 mM		NaCl 50 mM		NaCl 100 mM	
	Rel %	g/Kg	Rel %	g/Kg	Rel %	g/Kg	Rel %	g/Kg
*β*‐Pinene	0.22%d	1.65 ± 0.55	0.39%b	1.75 ± 0.25	0.51%a	2.55 ± 0.39	0.31%c	0.59 ± 0.37
Sabinene	0.07%d	0.55 ± 0.43	0.21%b	0.96 ± 0.56	0.10%c	0.51 ± 0.05	0.27%a	0.50 ± 0.24
Limonene	0.36%b	2.73 ± 0.41	0.31%c	1.41 ± 0.24	0.37%b	1.86 ± 0.07	0.51%a	0.96 ± 0.15
1,8‐Cineole	7.60%c	57.89 ± 3.08	7.80%b	35.00 ± 1.95	7.82%b	38.86 ± 2.41	10.33%a	19.43 ± 2.96
Menthone	47.93%a	364.99 ± 2.28	31.23%c	140.2 ± 6.65	36.44%bc	181.05 ± 20.32	38.00%b	71.46 ± 14.36
*E*‐Sabinene hydrate	2.83%c	21.55 ± 0.58	2.94%b	13.21 ± 0.14	2.78%c	13.79 ± 0.15	3.63%a	6.83 ± 0.71
Menthofuran	7.14%c	54.33 ± 0.38	20.70%a	92.95 ± 8.00	17.31%b	86.04 ± 0.63	17.43%b	32.79 ± 15.37
Isomenthone	5.20%a	39.61 ± 0.62	2.85%v	12.79 ± 0.08	2.73%v	13.57 ± 1.37	3.83%b	7.21 ± 1.38
Linalool+isopulegone	1.00%ab	7.59 ± 0.11	0.80%b	3.61 ± 0.43	1.08%ab	5.38 ± 0.66	1.19%a	2.23 ± 0.56
*β*‐Cubebene	0.65%a	4.92 ± 0.14	0.54%b	2.43 ± 0.03	0.50%b	2.47 ± 0.35	0.65%a	1.22 ± 0.33
*E*‐*β*‐caryophyllene	2.94%b	22.38 ± 0.05	1.69%c	7.61 ± 0.77	3.33%a	16.56 ± 2.84	3.46%a	6.51 ± 1.69
Neomenthol	0.53%d	4.00 ± 0.02	0.73%c	3.26 ± 0.09	1.27%b	6.30 ± 0.28	2.05%a	3.85 ± 0.73
Pulegone	6.19%d	47.16 ± 0.16	16.58%a	74.44 ± 4.86	10.33%b	51.32 ± 3.78	7.17%c	13.48 ± 5.78
Menthol	4.59%c	34.94 ± 0.22	2.39%d	10.73 ± 0.45	7.47%b	37.13 ± 5.37	10.33%a	19.42 ± 1.09
*ε*‐Muurolene	0.26%b	1.95 ± 0.02	0.10%c	0.43 ± 0.05	0.30%a	1.49 ± 0.21	0.32%a	0.60 ± 0.14
*α*‐Humulene	0.17%	1.30 ± 0.06	0.00%	0.00 ± 0.00	0.14%	0.69 ± 0.02	0.16%	0.31 ± 0.11
*Z*‐Muurola‐4 (15), 5‐diene	0.20%a	1.56 ± 0.11	0.10%b	0.45 ± 0.07	0.14%b	0.72 ± 0.01	0.13%b	0.24 ± 0.09
(*Z*)‐*β*‐Farnesene	0.24%c	1.81 ± 0.10	0.37%b	1.65 ± 1.01	0.88%a	4.35 ± 0.48	0.91%a	1.71 ± 0.40
*γ*‐Muurolene	0.12%	0.93 ± 0.08	0.10%	0.47 ± 0.06	0.09%	0.44 ± 0.06	0.11%	0.21 ± 0.04
Ledene	0.13%a	0.99 ± 0.13	0.05%b	0.21 ± 0.03	0.06%b	0.30 ± 0.01	0.06%b	0.12 ± 0.03
Germacrene D	4.36%a	33.23 ± 0.89	2.48%b	11.13 ± 1.64	4.60%a	22.88 ± 3.23	4.77%a	8.97 ± 2.61
*α*‐terpineol	0.49%c	3.76 ± 0.61	0.79%a	3.54 ± 0.24	0.56%b	2.79 ± 0.09	0.54%b	1.01 ± 0.72
Piperitone	0.94%ab	7.18 ± 0.35	0.55%c	2.47 ± 0.06	0.81%b	4.04 ± 0.52	1.03%a	1.93 ± 0.41
Bicyclogermacrene	0.42%b	3.17 ± 0.14	0.18%c	0.81 ± 0.05	0.43%b	2.14 ± 0.35	0.47%a	0.88 ± 0.26
*γ*‐Cadinene	0.10%a	0.78 ± 0.07	0.04%b	0.17 ± 0.02	0.06%b	0.30 ± 0.01	0.06%b	0.12 ± 0.02
*δ*‐Cadinene	0.17%a	1.31 ± 0.05	0.07%b	0.32 ± 0.01	0.17%a	0.83 ± 0.11	0.12%ab	0.23 ± 0.06
Cubebol	0.14%b	1.09 ± 0.05	0.11%b	0.48 ± 0.02	0.21%a	1.05 ± 0.12	0.20%a	0.38 ± 0.10
Viridiflorol	0.98%a	7.46 ± 0.31	0.55%b	2.46 ± 0.18	1.13%a	5.63 ± 0.81	1.06%a	1.99 ± 0.48
Total yield		761.44 ± 17.47		448.98 ± 55.36		496.92 ± 89.29		188.07 ± 4.99

*Note:* Lowercase letters indicate statistical differences among the samples, as calculated by one‐way ANOVA followed by Tukey's post hoc test (*p* < 0.01). ±standard deviation. Rel. %, relative percentage.

The terpenoid profile of peppermint cuttings is characterized by a high content of menthone and pulegone, which serve as precursors to the main compound, menthol. In line with the overall reduction in terpenoid content, most individual compounds exhibited significant reductions under salt stress (Table 1). The most pronounced changes involved menthone, which decreased sharply from 364.99 ± 2.28 g/kg in the control to 71.46 ± 14.36 g/kg at 100 mM NaCl, isomenthone (reduced from 39.61 ± 0.62 to 7.21 ± 1.38 g/kg), menthol (decreased from 34.94 ± 0.22 to 19.42 ± 1.09 g/kg), and 1,8‐Cineole (that dropped from 57.89 ± 3.08 to 19.43 ± 2.96 g/kg). In contrast, pulegone and menthofuran both showed an initial increase at intermediate salt concentrations before declining at 100 mM. Pulegone reached 74.44 ± 4.86 g/kg at 25 mM NaCl before declining to 13.48 ± 5.78 g/kg at 100 mM. Similarly, menthofuran showed an initial increase (from 54.33 ± 0.38 to 92.95 ± 8.00 g/kg at 25 mM) followed by a reduction at 100 mM NaCl (32.79 ± 15.37 g/kg). The relatively high baseline levels of pulegone and menthofuran observed in the control plants likely reflect the natural developmental stage of essential oil biosynthesis in *Mentha × piperita* at the time of sampling, during which the conversion of these precursors to menthol is not yet fully active.

To better understand the effects of salt stress on the terpenoid profile, we performed a cluster analysis using the quantitative data of Table 1. The analysis clearly separated control samples from treated plants (Figure 1B). As expected, the 50 and 100 mM treatments were closely grouped, indicating a similar modulation pattern of terpenoids, whereas the 25 mM treatment showed a terpenoid modulation pattern like the controls, reflecting a more moderate metabolic response.

To identify which compounds contributed most to the observed clustering, we performed a principal component analysis (PCA) using rotated loadings with varimax variation (Figure 1C) to improve factor interpretability. Factor 1, which accounted for 45% of the total variance, separated the major compounds, including pulegone, menthofuran, menthone, and menthol. The two main sesquiterpenes, germacrene D and *β*‐caryophyllene, were separated by negative Factor 1 and positive Factor 2 scores, with Factor 2 explaining an additional 44% of total variance. Finally, neomenthol and other minor compounds were separated by negative Factor 1 and Factor 3 scores, with Factor 3 accounting for 10% of the variance.

**FIGURE 2:**
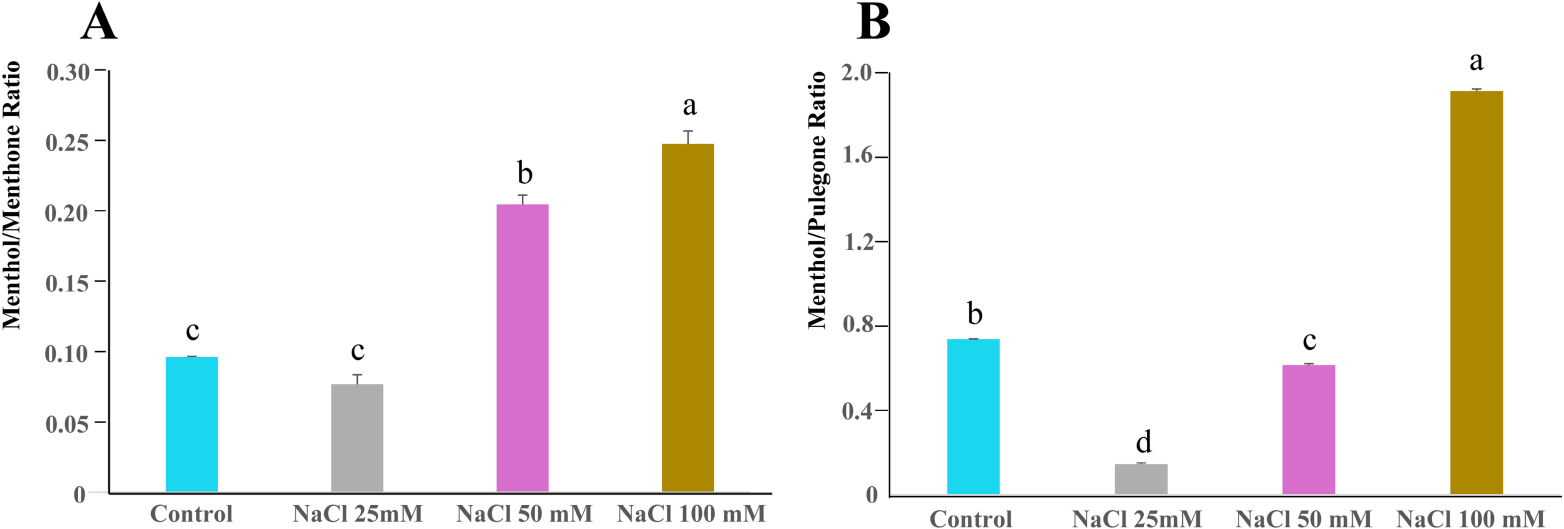
Response of menthol to increasing NaCl concentrations. (A) Ratio of menthol to menthone. (B) Ratio of menthol to pulegone. For both panels, histograms represent the mean values of three different biological replicates, while error bars indicate the standard deviation. Different letters above the bars denote a significant difference (*p* < 0.05) between treatments, as calculated by one‐way ANOVA followed by Tukey's post hoc test.

We next evaluated the impact of salt stress on menthol production and its biosynthetic pathway. The menthol‐to‐menthone ratio, indicative of conversion efficiency by menthone reductase, increased significantly at 50 and 100 mM NaCl (Figure 2A). This suggests an enhanced conversion of menthone to menthol under high salt stress conditions. Conversely, the ratio of menthol to pulegone (the precursor to menthone) showed a more complex response. This ratio decreased at 25 and 50 mM NaCl, indicating a reduction in the overall flux from pulegone to menthol. However, a significant increase was found in plants treated with 100 mM NaCl (Figure 2B). These results suggest a biphasic regulation of the pathway, where early steps in the pathway may be inhibited under moderate stress levels, while compensatory mechanisms may be activated under severe stress. Raw data for the terpenoid analysis are provided in Supporting Information S1.

### 3.2 Salt Stress Modulates Genes Involved in Peppermint Terpenoid Biosynthesis

**FIGURE 3:**
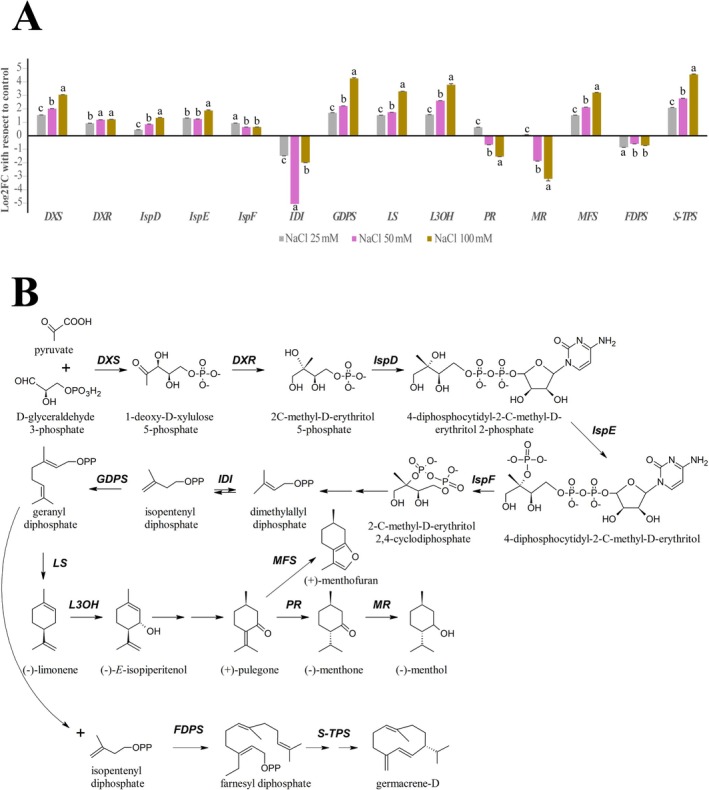
Effects of salt stress on peppermint terpenoid biosynthesis genes. (A) Gene expression analysis of terpenoid pathway genes. Log_2_ fold change values, calculated relative to control plants and normalized to the reference gene *UBQ*, are shown for various genes involved in peppermint terpenoid biosynthesis. (B) Schematic of the menthol biosynthetic pathway and associated genes. See text for gene nomenclature. Molecule structure and gene nomenclature from Perez‐Gil et al. (2024) and Johnson et al. (2017).

To determine whether the changes in terpenoid production were due to altered gene expression, we used quantitative PCR to assess the transcript levels of key biosynthetic genes. Surprisingly, most of the analysed genes showed an upregulation trend with increasing NaCl concentration (Figure 3A), despite the overall decrease in terpenoid yield.

All genes in the MEP pathway were upregulated. Notably, 1‐deoxy‐d‐xylulose‐5‐phosphate synthase (*DXS*), which catalyses the initial step of converting pyruvate and d‐glyceraldehyde 3‐phosphate to 1‐deoxy‐d‐xylulose 5‐phosphate, showed an increasing expression trend (Figure 3B). In contrast, a strong, hormetic downregulation was observed for isopentenyl diphosphate isomerase (*IDI*), which is responsible for the isomerization of the C_5_ precursors, isopentenyl diphosphate and dimethylallyl diphosphate (Figure 3A,B). The three genes responsible for building the menthol skeleton [geranyl diphosphate synthase (*GDPS*), (−)‐limonene synthase (*LS*), and (−)‐limonene‐3‐hydroxylase (*L3OH*)] all showed similar upregulation trends with increasing NaCl concentration (Figure 3A,B). However, genes coding for the final steps of menthol production were downregulated. (+)‐Pulegone reductase (*PR*), the enzyme responsible for menthone production, was slightly upregulated at 25 mM NaCl but progressively downregulated at higher concentrations. Similarly, (−)‐menthone/(−)‐menthol reductase (*MR*), the final enzyme in the menthol pathway, showed a strong downregulation trend, especially as NaCl concentrations increased (Figure 3A,B). In parallel, (+)‐menthofuran synthase (*MFS*), which forms the furan ring of menthofuran from pulegone, and a putative sesquiterpene synthase (*S‐TPS*), involved in germacrene‐D formation, were both increasingly upregulated with increasing NaCl concentration. Conversely, farnesyl diphosphate synthase (*FDPS*), an enzyme that generates farnesyl diphosphate for sesquiterpene biosynthesis, was downregulated by salt stress without a clear dose‐dependent trend (Figure 3A). Raw gene expression data are available in Supporting Information S2.

### 3.3 Salt Stress Alters Peppermint Photosynthetic Pigments and Photosystem II Photochemistry and Efficiency

**FIGURE 4:**
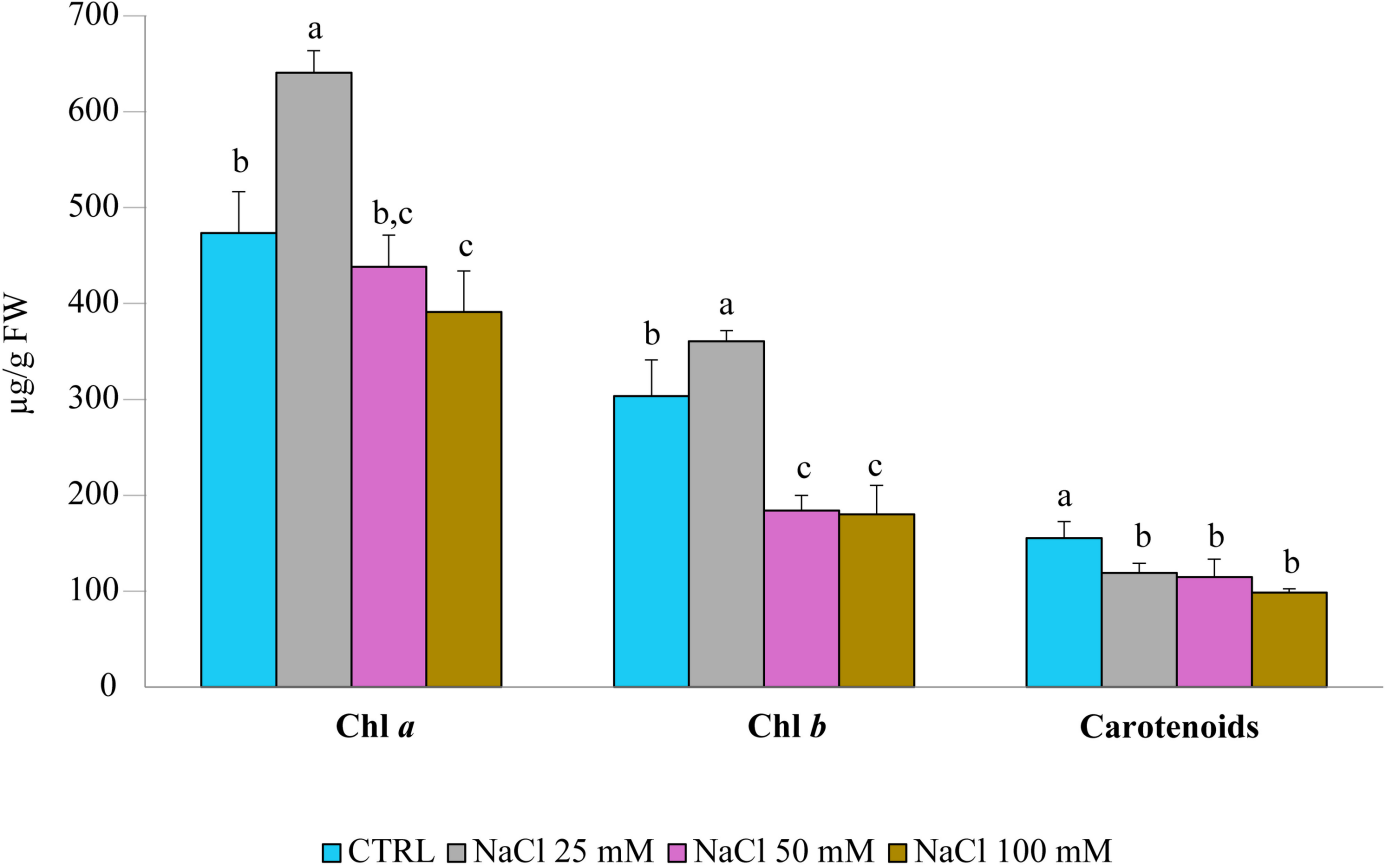
Chlorophyll and carotenoid content of peppermint as a function of NaCl concentration. Histograms represent the mean values of three different biological replicates, while error bars indicate the standard deviation. Different letters above the bars denote a significant difference (*p* < 0.05) between treatments, as calculated by one‐way ANOVA followed by Tukey's post hoc test.

Terpenoids biosynthesis directly depends on triose phosphates produced during photosynthesis, making this process particularly vulnerable to stress‐induced changes in the photosynthetic machinery. Given the known impact of salt stress on chloroplast function, we investigated its effect on peppermint leaf pigments. Our analysis revealed a significant increase in both chlorophyll *a* (Chl *a*) and chlorophyll *b* (Chl *b*) content in plants treated with 25 mM NaCl compared to controls (Figure 4). However, as the NaCl concentration increased, pigment levels were negatively affected. Chl *b* content was significantly reduced by both 50 and 100 mM NaCl treatments, while a significant reduction in Chl *a* was observed only at the highest concentration of 100 mM NaCl. Carotenoid content was consistently reduced by all NaCl treatments (Figure 4). Raw data for chlorophyll and carotenoid measurements are available in Supporting Information S3.

**FIGURE 5:**
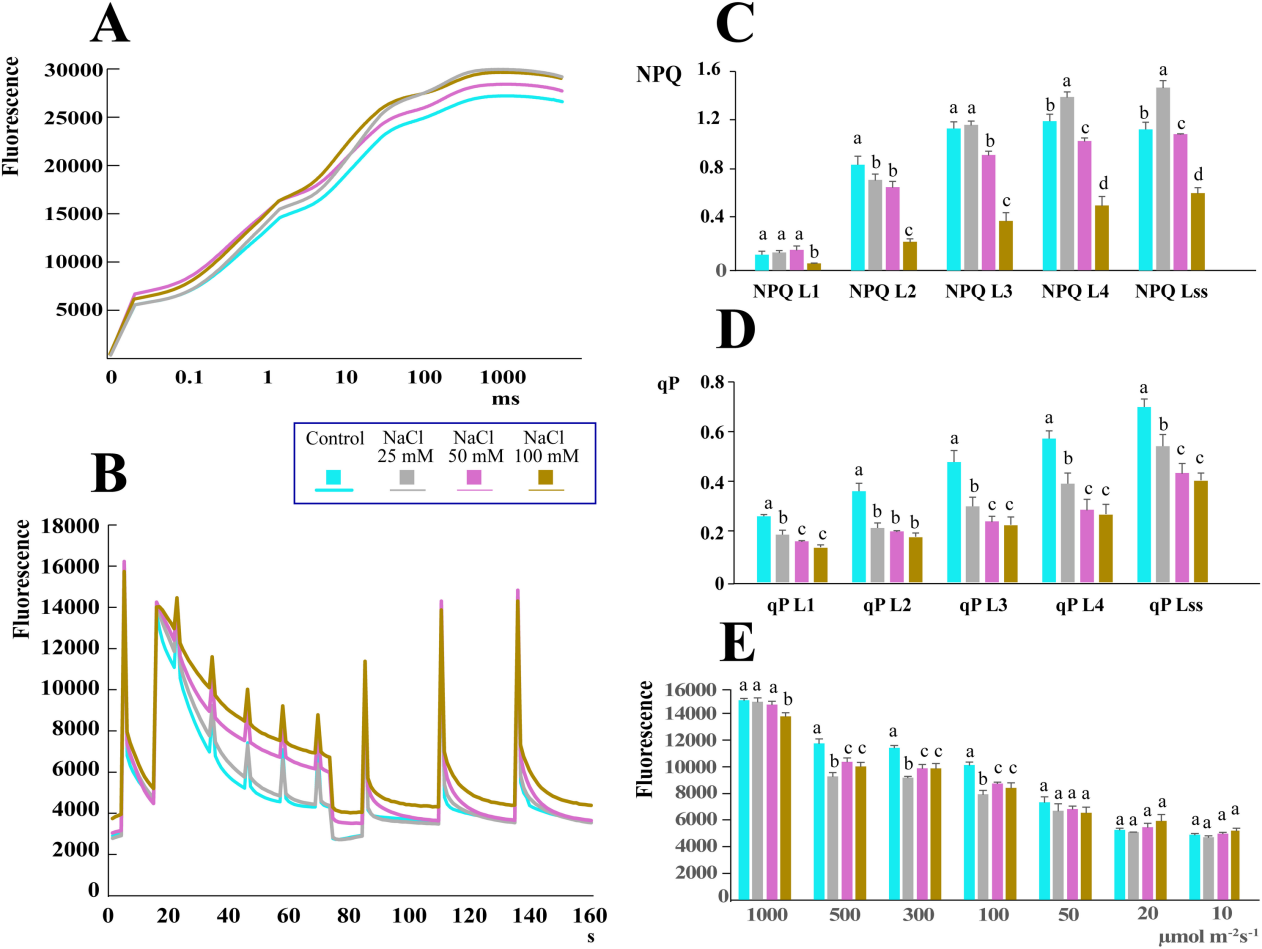
Impact of salt stress on peppermint photosynthesis. (A) OJIP representation of chlorophyll fluorescence. The curves represent the average of at least three measurements per treatment. (B) Non‐photochemical quenching (NPQ) at varying light intensities illustrates the capacity of peppermint to dissipate excess light energy as heat. (C) Coefficient of photochemical quenching (qP). This coefficient provides an estimate of the proportion of open PSII reaction centres. (D) Light response changes in photosynthetic parameters. This panel shows the quantum yield of photosynthesis as a function of light intensity, measured by pulse amplitude modulated (PAM) fluorometry. (E) Quantum yield of photosynthesis under continuous illumination. This graph shows the overall efficiency of photosynthesis at different light intensities. Histograms represent the mean values of three different biological replicates, while error bars indicate the standard deviation. Different letters above the bars denote a significant difference (*p* < 0.05) between treatments, as calculated by one‐way ANOVA followed by Tukey's post hoc test.

To evaluate the integrity and efficiency of PSII photochemistry, we measured OJIP fluorescence transients. As shown in Figure 5A, increasing NaCl concentrations resulted in elevated fluorescence compared to controls. Significant differences were observed for the Area (representing the size of the area between the fluorescence curve and *F*_m_) and *S*_s_ [the smallest Area/(*F*_m_—*F*_0_) single turn‐over] parameters, both of which decreased progressively with salinity, indicating an inhibition of electron transport (see Supporting Information S4).

We then analysed key fluorescence quenching parameters to understand how light energy is utilized and dissipated by peppermint leaves under salt stress. Figure 5B shows the fluorescence kinetics of peppermint leaves from control and treated plants under actinic light. Fluorescence progressively increased with rising NaCl concentrations, suggesting a decrease in photochemical efficiency. Non‐photochemical quenching (NPQ), a mechanism for dissipating excess light energy as heat, was measured at different light intensities (from NPQ L1 to NPQ L4) (Figure 5C). At the initial light level (L1), significant differences were found only at the highest NaCl concentration (100 mM). As light intensity increased (L2–L4), NPQ became significantly lower in plants treated with 50 mM NaCl compared to controls. Interestingly, plants treated with 25 mM NaCl showed a progressive increase in NPQ relative to controls across all light levels. The NPQ response at steady‐state illumination (NPQ Lss) was like that observed at the highest light intensity.

Photochemical quenching (qP), an indicator of the proportion of open (oxidized) PSII reaction centers indicating the efficiency of electron transport, was consistently reduced in all salt‐treated plants compared with controls across all light levels and at steady‐state (Figure 5D). Similar results were found for the quantum yield of PSII (QY), as detailed in Supporting Information S4.

Finally, we assessed the quantum yields of photosynthesis under various light intensities using a pulse‐modulated fluorometer (PAM) (Figure 5E). At the highest illumination (1000 μmol m^−2^ s^−1^), a significant reduction was found only for plants treated with 100 mM NaCl. However, at intermediate light intensities (500 and 100 μmol m^−2^ s^−1^), fluorescence was significantly reduced in plants treated with all NaCl concentrations. No significant differences were observed between control and treated plants at lower light intensities. Raw fluorescence data are reported in Supporting Information S4.

### 3.4 Peppermint Modulates Flavonoid Production and Chalcone Isomerase Expression in Response to Salt Stress

**FIGURE 6:**
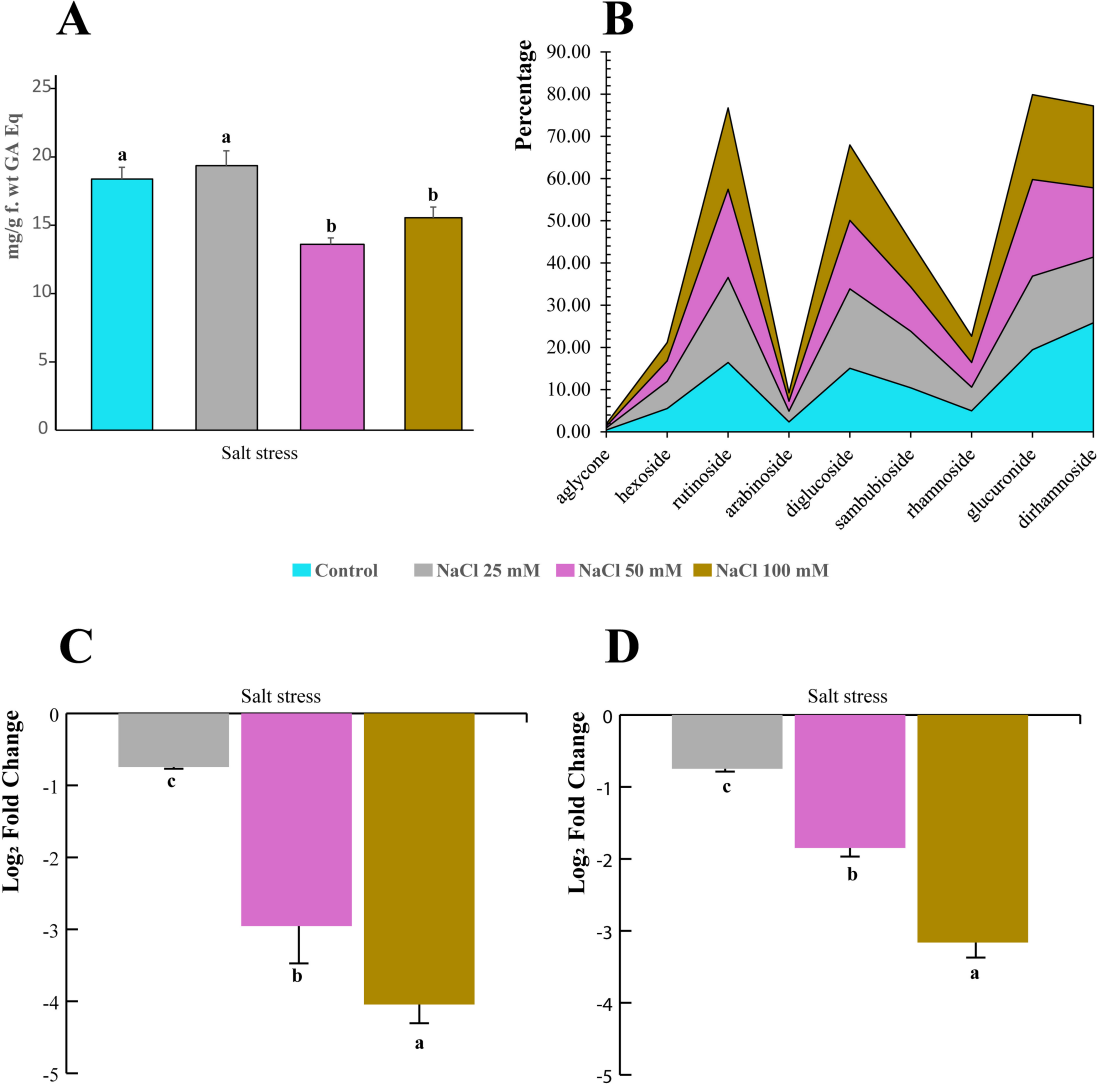
Impact of salt stress on peppermint phenolic and flavonoid content (A) Concentration of total phenolic compounds in peppermint leaves exposed to increasing concentrations of NaCl. (B) Relative abundance of various flavonoid conjugates in response to increasing NaCl concentrations. (C, D) Relative expression levels of two Chalcone‐flavanone Isomerase (*CHI*) homologs (AT3G55120 homolog in C and AT5G05270 homolog in D) in peppermint under different NaCl treatments. Histograms represent the mean values of three independent biological replicates, and error bars indicate the standard deviation (SD). Different letters above the bars denote a significant difference (*p* < 0.05) between treatments, as determined by one‐way ANOVA followed by Tukey's post hoc test.

In addition to terpenoids, phenolic compounds play a crucial role in peppermint's strategy to cope with abiotic stress. Our analysis showed that the total phenolic content was significantly reduced in plants exposed to 50 and 100 mM NaCl compared to controls. In contrast, the 25 mM NaCl treatment did not produce a statistically significant change (Figure 6A). Interestingly, this overall reduction in total phenols was accompanied by a qualitative and quantitative change in the flavonoid profile, as determined by LC–MS. Specifically, the content of various conjugated flavonoid aglycones (including those with rutinoside, diglucoside, sambubioside, glucuronide, and dirhamnoside conjugations) increased with rising NaCl concentration (Figure 6B). Raw data for total phenolics and the LC–MS analysis are provided in Supporting Information S5 and S6, respectively.

While the spectrophotometric quantification of total phenols (Figure 6A) provided an overview, LC–MS analysis offered a more precise pattern of flavonoid responses in peppermint to NaCl stress. In particular, polyphenol levels at 25 mM NaCl remained comparable to the control, with a progressive decrease observed at higher salt concentrations (50 and 100 mM). Consistent with these findings, we assessed the gene expression of two Chalcone‐flavanone isomerase (CHI) homologs from the Arabidopsis family proteins (homologs of AT3G55120 and AT5G05270, respectively). The expression of the two *CHI* homologs was markedly downregulated under increasing salt stress, which is consistent with the suppression of flavonoid biosynthesis at elevated salinity levels (Figure 6C,D).

**FIGURE 7:**
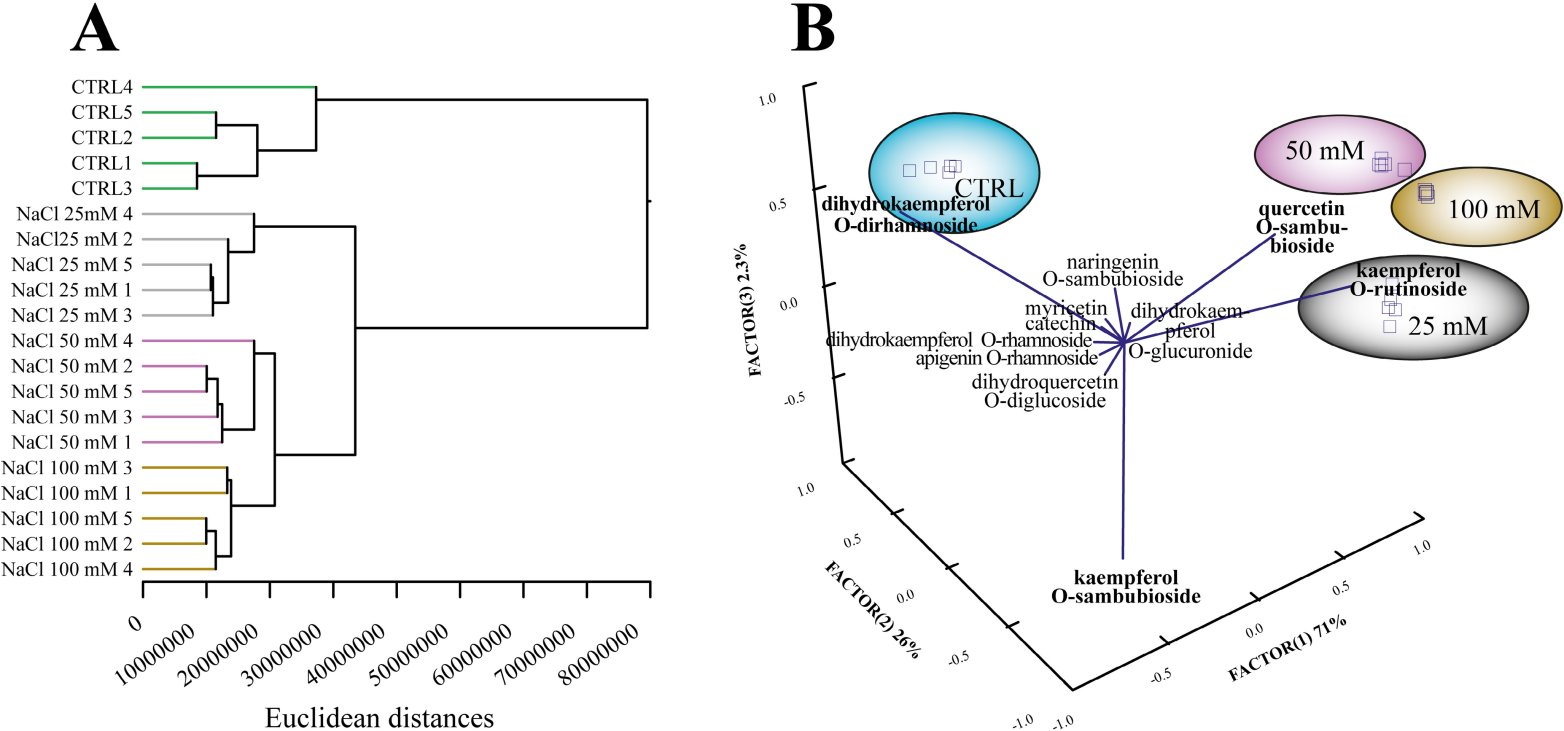
Modulation of peppermint flavonoid glycosides in response to salt stress. (A) Euclidean cluster analysis of flavonoid profiles. The dendrogram was constructed using the single linkage method and Euclidean distances allowing the distinct separation of the flavonoid profile between control plants and those exposed to NaCl. (B) Principal component analysis (PCA) of flavonoid profiles showing the distribution of individual flavonoid compounds relative to the plant's response to different NaCl concentrations.

Multivariate analysis of individual flavonoid compounds confirmed this response. A Euclidean cluster analysis performed on the different biological replicates showed a strong consistency within groups, with a clear separation between control and treated plants (Figure 7A). Among the treatments, a close Euclidean distance was observed between the 50 and 100 mM NaCl groups, indicating a similar flavonoid profile under these conditions (Figure 7A).

The PCA provided additional resolution on the patterns detected in the flavonoid data by identifying the compounds most responsible for the observed clustering (Figure 7B). Factor 1 scores separated kaempferol‐O‐rutinoside and quercetin‐O‐sambubioside from the other compounds. Kaempferol‐O‐sambubioside, on the other hand, was separated by negative Factor 1 and Factor 2 scores, respectively. Conversely, dihydrokaempferol‐O‐dirhamnoside was separated by positive Factor 3 scores and was closely associated with the control samples. A heatmap analysis, with clustering performed using Pearson distances and single linkage, showed a clear modulation between control and NaCl‐exposed plants (Figure S2). A strong downregulation was observed for most of the flavonoid indicators upon 100 mM NaCl exposure, which contrasts with the distinct upregulation trend observed for genes involved in terpenoid expression (Figure S2).

## 4 Discussion

Salt stress is a major abiotic stressor that significantly limits crop production globally, particularly in arid and semi‐arid regions (Fu and Yang 2023). It negatively affects plant growth and development by disrupting water uptake, causing ion toxicity, and inducing stress. For aromatic and medicinal plants like peppermint (*Mentha* × *piperita* L.), which is highly valued for its essential oil, salt stress not only impacts biomass but also alters the yield and composition of secondary metabolites, such as terpenoids and phenolics, which are the source of its commercial value (Kumar et al. 2023). The intricate mechanisms by which peppermint responds to salt stress are not fully understood. Our results suggest that the decreased production of terpenoids is directly linked to an impaired photosynthetic capacity, while the plant's metabolic resources are simultaneously reallocated toward a highly targeted flavonoid defence.

### 4.1 Terpenoid Production and Gene Expression Evidence a Central Paradox

Previous studies have consistently shown that salinity can significantly impact both essential oil yield and composition of secondary metabolites in *Mentha* species, including peppermint, often at NaCl concentrations comparable to those tested in this work (Aziz et al. 2008; Oueslati et al. 2010; Çoban 2016). In peppermint, yield reduction under salt stress has been previously demonstrated, underscoring both the economic relevance of the problem and the importance of elucidating its mechanistic basis (Cappellari et al. 2020; Zhao, Wei, et al. 2022). In particular, like other abiotic stresses, salt stress impacts the production of plant secondary metabolites, including terpenoids (Ramakrishna and Ravishankar 2011).

Our results show a significant reduction in terpenoid yield despite the general upregulation of most biosynthetic genes. This study's findings on gene regulation are partly consistent with and partly diverge from other reports. For instance, Profico et al. (2025) reported that NaCl exposure can lead to a reduction in menthone while relatively increasing menthol content in peppermint, in contrast with our observations. Our data showed upregulation of *IDI*, *GDPS*, and *LS* in response to NaCl, while a previous study found no significant regulation of these genes under similar stress conditions (Zhao, Wei, et al. 2022), although the consistent downregulation of *PR* aligns with our observations. The paradoxical transcription–metabolite disconnection of terpenoid biosynthesis is highlighted by our data. For example, genes like *GDPS* and *LS* showed strong upregulation, with Log_2_ fold change values reaching 4.25 and 3.29 at 100 mM NaCl, respectively. However, this transcriptional activation did not translate to an increase in overall product, as the total terpenoid content decreased simultaneously. Conversely, the reduction in menthol and menthone can be directly attributed to the significant downregulation of the final two enzymes in the pathway, *PR* and *MR*, with *MR* showing a particularly strong negative fold change at 100 mM NaCl.

The variable behaviour of metabolites under different salt concentrations likely reflects complex and non‐linear metabolic responses. For example, the observed drop at 25 mM NaCl may represent an early stress‐induced inhibition of specific terpenoid pathways, whereas the partial increase at 50 mM could be due to stress‐adaptive metabolic reprogramming, where the plant reallocates carbon and precursors to maintain key metabolites. The marked upregulation of *MFS* at 100 mM NaCl suggests a redirection of metabolic flux away from the menthol pathway towards the production of menthofuran, potentially as part of a stress‐specific defence mechanism, consistent with its role as a stress‐related molecule in *Mentha aquatica* (Zebelo et al. 2011). The paradox can be attributed to several factors. The lack of correlation between high gene expression and low product yield suggests that transcriptional upregulation may not be the sole determinant of flux; post‐transcriptional or post‐translational regulation could occur, making enzymes non‐functional or leading to their rapid degradation (Zelezniak et al. 2014). Furthermore, as we will discuss, photosynthetic impairment likely results in a severe limitation of triose phosphate precursors, starving the pathway of its raw materials despite the active transcription of upstream genes.

### 4.2 Potential Substrate Limitation due to Impaired Photosynthetic Efficiency

While our transcriptional data indicate upregulation of much of the terpenoid biosynthetic machinery, the overall decline in product yield under salt stress is most plausibly explained by a limitation in photosynthetic efficiency. Indeed, salt stress impaired photosynthesis, reducing both pigment content and PSII efficiency. Photosynthesis provides the triose phosphates that are the fundamental building blocks for the MEP pathway, which produces terpenoids (Perez‐Gil et al. 2024). Consequently, in the absence of sufficient carbon skeletons, plant glandular trichomes are unable to sustain high rates of terpenoid biosynthesis despite elevated transcript levels. This indicates a severe energy deficit and a broader stress‐driven reallocation of metabolic resources, as a lack of photosynthetic products forces the plant to prioritize survival over secondary metabolite synthesis. Our data on fluorescence parameters directly evidence this photosynthetic impairment. A reduced quantum yield, especially at higher light intensities, indicates that a smaller fraction of the absorbed light energy is being used productively for photochemistry. Simultaneously, the consistently low photochemical quenching (qP) in all salt‐treated plants shows that PSII reaction centres are more closed (Roháček 2002). This is a result of an inhibited or slowed electron transport chain, which prevents the conversion of light energy into chemical energy. Furthermore, the decrease in non‐photochemical quenching (NPQ) at higher stress levels (50–100 mM NaCl) indicates a critical failure of the plant's protective mechanisms to safely dissipate excess energy as heat (Zuo 2025). A reduction in photosynthetic efficiency in peppermint was also found under salt stress (Tabatabaie and Nazari 2007). Taken together, these data indicate a photosynthetic system under severe stress, struggling to convert absorbed light energy into the chemical energy necessary for survival and growth.

### 4.3 Salt Stress Prompts a Metabolic Shift to a Coordinated Defence

Salt stress caused a pronounced alteration in peppermint's phenolic metabolism, marked by a decrease in total phenolic content alongside an increase in specific flavonoid conjugates. While the reduction in the total amount of phenolics may reflect limited carbon availability or stress‐related degradation to save energy, the significant accumulation of specific flavonoid conjugates (i.e., kaempferol‐ and quercetin‐glycosides) highlights a strategic investment of limited resources in certain branches of the flavonoid pathway that are maintained or even favoured under stress. These compounds are highly effective antioxidants and osmo/photoprotectants, and their glycosylation likely enhances their solubility, stability, and antioxidant capacity, making them more effective at scavenging toxic compounds (Tang et al. 2024). This targeted production serves a critical function: to protect the remaining photosynthetic machinery from light‐induced damage by acting as a form of internal sunscreen (Saewan and Jimtaisong 2013).

To better understand physiological changes in secondary metabolism of peppermint plants, it should be considered that flavonoids and terpenes are synthesized through distinct biosynthetic pathways. While the shikimate pathway (starting from aromatic amino acids such as phenylalanine and tyrosine) follows in the biosynthesis of flavonoids, the MEP pathway achieves the formation of terpenes. However, these pathways are metabolically interconnected through the sharing of carbon pools and precursors derived from photosynthesis. Under conditions of salt stress, this interconnection becomes particularly evident, as the plant reallocates carbon flow between these major metabolic networks to optimize defence and energy balance. In our experimental setup, the flavonoid response runs parallel to the terpenoid response, indicating that secondary metabolism is not generally suppressed under salt stress. Instead, peppermint exhibits a complex reorganization of its metabolic outputs under constrained conditions. Reduced photosynthetic capacity likely limits oxidative damage but also limits carbon availability, contributing to a decline in total phenolics and menthol‐related terpenoid. In contrast, menthofuran and specific glycosylated flavonoids increase, suggesting that certain metabolic branches remain active or are selectively maintained. The simultaneous modulation of the terpenoid and flavonoid branches therefore reflects integrated metabolic reprogramming rather than independent adjustments. The reduction in terpenes parallels the decrease in the availability of triose phosphates, while the selective overproduction of specific flavonoid conjugates suggests a priority in the synthesis of highly efficient antioxidant defences. This balance indicates a coordinated metabolic strategy aimed at maintaining redox homeostasis and photoprotection during salt stress.

### 4.4 An Integrated Model of Peppermint's Salt Stress Response

**FIGURE 8:**
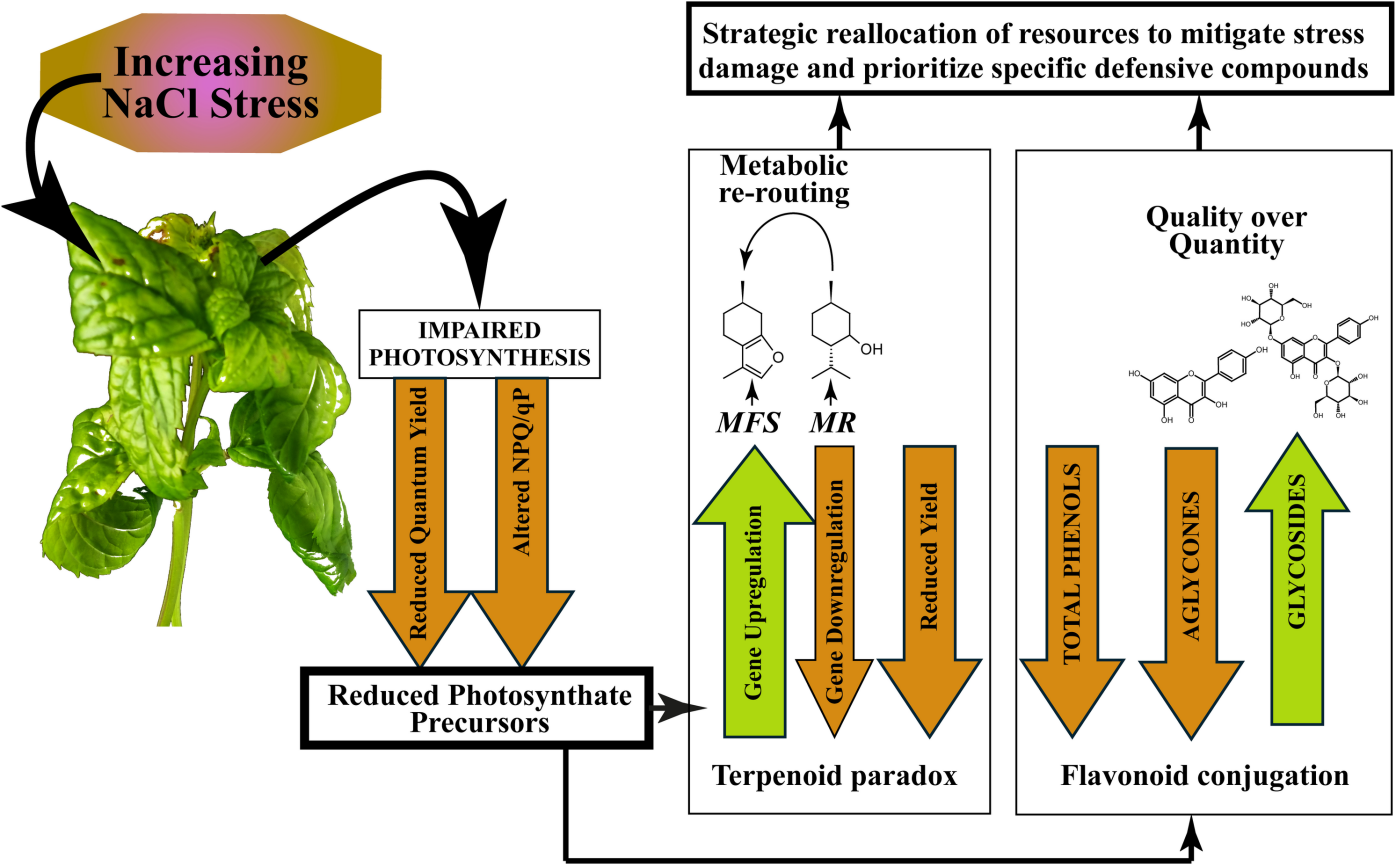
Integrated model of peppermint's metabolic response to increasing salt stress. Coordinated physiological and metabolic changes are observed in peppermint under salt stress. Increasing NaCl concentrations lead to impaired photosynthesis, which in turn causes a limitation of precursor molecules (e.g., triose phosphates). This limitation drives a metabolic reallocation. In the terpenoid pathway, genes encoding upstream enzymes are upregulated while the final steps leading to menthol are downregulated, creating a strategic bottleneck and redirecting metabolic flux toward menthofuran. Concurrently, the plant's flavonoid defence shifts from general, high‐volume phenolic production to a targeted increase in specific flavonoid conjugates, which serve as specialized antioxidants and photoprotectants. This integrated response demonstrates the plant's strategic management of compromised resources for survival.

Changes in the composition of terpenoids and phenolic compounds induced by salt stress can affect both the yield and quality of peppermint essential oils, with direct effects on the flavour, aroma, and commercial value of the products. In particular, based on the results, we propose a unified model for peppermint's responses to salt stress (Figure 8). At 25 mm NaCl, plants show a moderate stress response, characterized by upregulation of terpenoid biosynthetic genes, maintenance of relatively high terpenoid output, and increased production of specific photosynthetic pigments and flavonoids. This phase likely reflects partial compensation for emerging stress while core metabolic functions remain largely intact, thus contributing to the preservation of essential oil quality. Conversely, at higher NaCl levels (50–100 mM NaCl), the salt concentration overwhelms the plant's protective mechanisms. Indeed, photosynthetic impairment leads to a severe limitation of the availability of carbon precursors for secondary metabolism. Although many genes involved in terpenoid synthesis remained upregulated, the terminal steps in menthol biosynthesis are inhibited, leading to a drastic reduction in this compound. In parallel, specific flavonoid conjugates accumulate, potentially reflecting either selective activation of stress‐associated branches or differential stability of these metabolites under saline conditions. These changes can affect both the sensory and commercial properties of peppermint oil and the potential antioxidant and health benefits of phenolic compounds, highlighting the practical implications for the cultivation and use of mint in environments prone to salinity.

## 5 Conclusions

Overall, our findings reveal that peppermint responds to salt stress by employing a sophisticated metabolic strategy by re‐routing resources and adjusting key metabolic pathways to cope with stress, even at the cost of its overall terpenoid yield. In particular, the plant manages its compromised resources through targeted adjustments rather than a general metabolic failure (Figure 8).

We observed a paradoxical transcription–metabolite disconnection in terpenoid biosynthesis: a significant reduction in overall yield despite the upregulation of most pathway genes. This is driven by both a strategic bottleneck (the downregulation of key enzymes for menthol synthesis) and a metabolic re‐routing toward other compounds like menthofuran. These changes occur alongside severe photosynthetic impairment, as indicated by reduced quantum yield and altered quenching parameters, which likely constrain the supply of carbon precursors for secondary metabolism. In parallel, the plant shows a highly adaptive defence strategy by shifting from a high‐volume production of general phenolic compounds to a targeted increase in specific, potent flavonoid conjugates, which likely serve as specialized antioxidants and osmo/photoprotectants.

These findings highlight a new, integrated understanding of peppermint's stress response. Based on our results, future research should focus on the post‐transcriptional and post‐translational regulation of key enzymes like *MR* to fully unravel the mechanisms behind this paradox. This foundational knowledge is critical for developing new cultivation strategies or using selective breeding to create salt‐tolerant peppermint varieties that can maintain their high yield of commercially valuable essential oil. Figure 8 summarizes peppermint responses to salt stress.

## Author Contributions

M.E.M. wrote the manuscript with inputs from G.M. and F.C. M.E.M. and G.M. analysed all the data and interpreted the results. F.C. performed qRT‐PCR analysis under the supervision of M.E.M. G.M. performed HPLC‐DAD‐ESI‐MS/MS analyses. All authors reviewed the manuscript.

## Funding

This work was supported by Università degli Studi di Torino, MAFFEI_RILO2024, MANNINO_RILO2024. Fondazione Cassa di Risparmio di Cuneo, MINTIS grant, ID: 79380, SIME: 2024.2493, MANG_CRC.

## Supporting information


**Supporting Information S1.** Terpenoid raw data.

**Supporting Information S2.** Gene expression raw data.

**Supporting Information S3.** Chlorophylls and Carotenoids raw data.

**Supporting Information S4.** OJIP—Photosynthesis raw data.

**Supporting Information S5.** Total phenols and cal curve raw data.

**Supporting Information S6.** Flavonoid raw data.

**Figure S1:** Plant treatments and control.

**Figure S2:** General heatmap analysis reveals specific trends in peppermint.

**Table S1:** Primers used in this work.

## Data Availability

All raw data from the results presented in this work are provided as Supporting Information.
