# Supplementary figures and images for: Salt Stress Re‐Routes Terpenoid and Flavonoid Metabolism in Peppermint ( Mentha × Piperita L.)
Source: Physiol Plant. 2025 Dec 18;177(6):e70694. doi: 10.1111/ppl.70694 (PMC12715299; doi:10.1111/ppl.70694)

Supplementary Figure S1 – Plant treatments and control

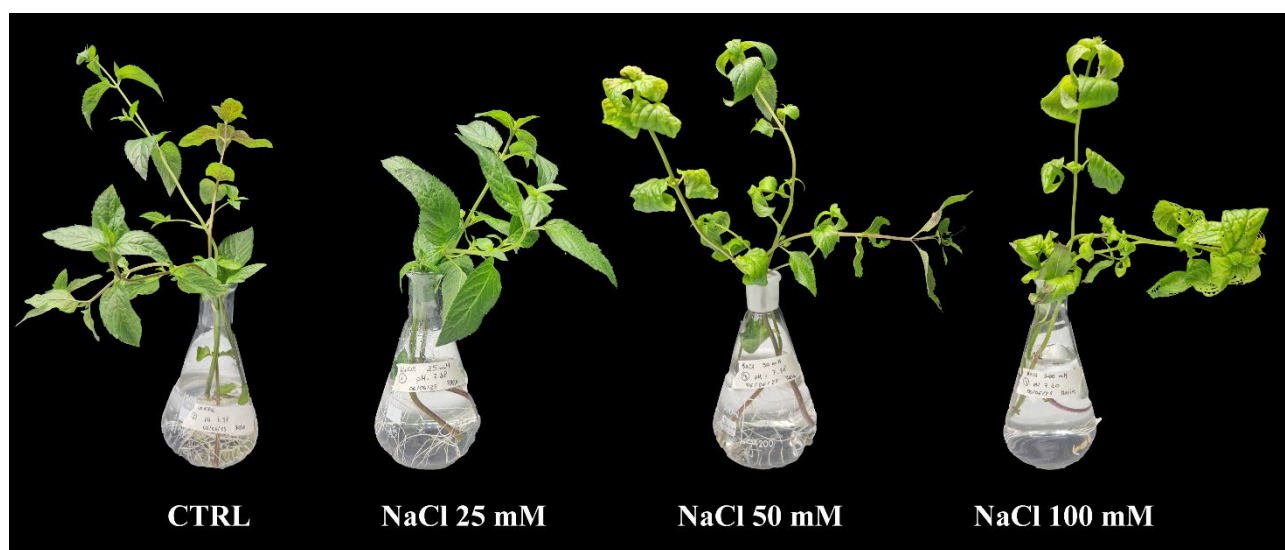

Supplement: Supplementary file 7 — Figure S1: Plant treatments and control. [file PPL-177-e70694-s007.pdf]
