# Supplementary material for: Salt Stress Re‐Routes Terpenoid and Flavonoid Metabolism in Peppermint ( Mentha × Piperita L.)
Source: Physiol Plant. 2025 Dec 18;177(6):e70694. doi: 10.1111/ppl.70694 (PMC12715299; doi:10.1111/ppl.70694)

Supplementary Figure S2. General heatmap analysis reveals specific trends in peppermint metabolic and gene expression responses to salt stress. Heatmap analysis with clustering performed with Pearson distances and single linkage. A clear modulation is observed between controls and plant exposed to NaCl. In general, a strong downregulation is observed for most of the indicators upon 100 mM NaCl exposure, with a clear and distinct trend for genes involved in terpenoid expression.

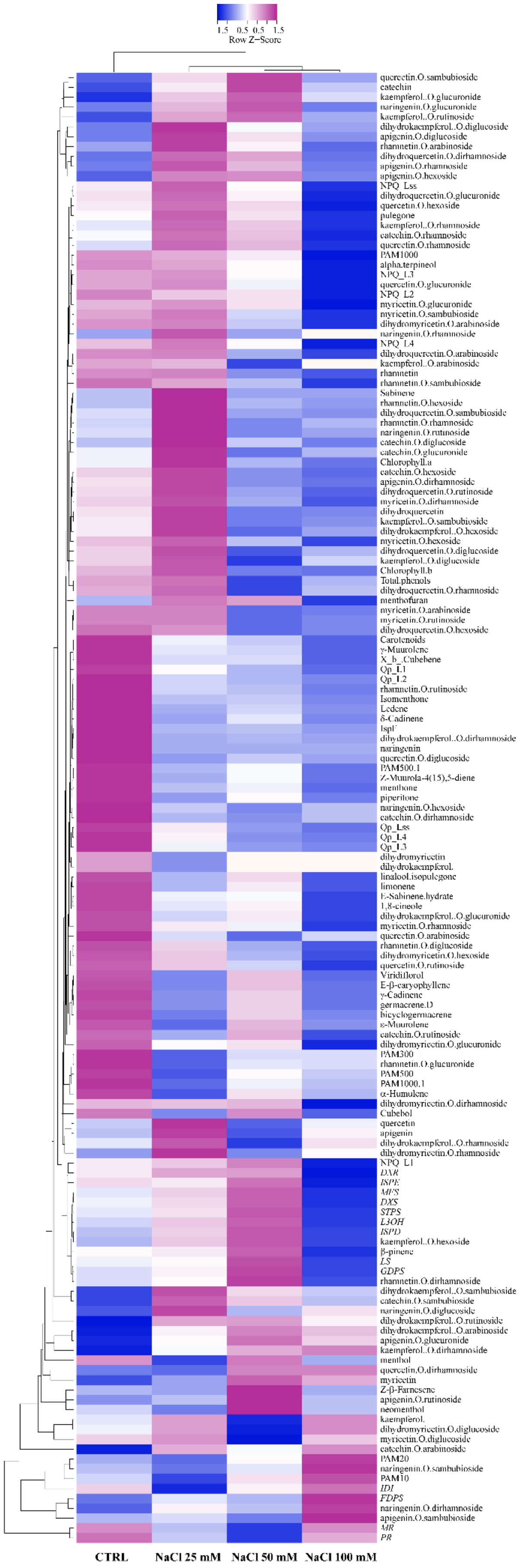

Supplement: Supplementary file 8 — Figure S2: General heatmap analysis reveals specific trends in peppermint. [file PPL-177-e70694-s006.pdf]
