# Supplementary material for: Salt Stress Re‐Routes Terpenoid and Flavonoid Metabolism in Peppermint ( Mentha × Piperita L.)
Source: Physiol Plant. 2025 Dec 18;177(6):e70694. doi: 10.1111/ppl.70694 (PMC12715299; doi:10.1111/ppl.70694)
Supplement: Supplementary file 9 — Table S1: Primers used in this work. [file PPL-177-e70694-s001.docx]

**Supplementary Table S1 – Primers used in this work**

| **Gene code** | **Description** | **Forward** | **Reverse** |
| --- | --- | --- | --- |
| *18S* | 18S ribosomal RNA | ATGATAACTCGACGGATCGC | CTTGGATGTGGTAGCCGTTT |
| *ACT* | Actin | GCACCCCATTCTCTTGACTGA | CGTCTCGAACATTATCTGAGTCAT |
| *DXR* | 1-Deoxy-D-xylulose 5-phosphate reductoisomerase | CGGCTACCTCGACATTTTCAA | GCGACACCGCCATTTCC |
| *DXS* | 1-Deoxy-D-xylulose 5-phosphate synthase | CCACCAGGCTTACCCACACAA | GCCACCGCCATCCCTAAAC |
| *FDPS* | Farnesyl-dihosphate synthase | GGAGAACCATCCAACTGTAA | GCCTTTACAACCAGCCAAGA |
| *GDPS* | Geranyl-diphosphate synthase | ATCTCAGCCGTTCTCCTTCA | CCTTATTGGGATGGATTTCT |
| *IDI* | Isopentenyl diphosphate isomerase | TCCCGCTCAACGAATTCACTCCAT | TCTGGGTTCGGATGAAGCTCAACA |
| *IspD* | 4-diphosphocytidyl-2-C-methyl-D-erythritol synthase | GAGCTTCTATGGGCAGGTCA | GGCCACGAATGGTAAACACT |
| *IspE* | 4-Diphosphocytidyl-2-C-methyl-D-erythritol 2-phosphate kinase | AGCCAATGGAGAAATGATCG | GAGAGGAATGAGGGCTTGTG |
| *IspF* | 2-C-methyl-D-erythritol 2,4-cyclodiphosphate synthase | TTGGAGTAGAGCCTCAGTCGG | CAACTTAGGATTGGTGTCGGG |
| *L3OH* | (-)-Limonene 3-hydroxylase | CCCCATCACCACCAACTCCA | GCTCCGCCAGCACCCATAG |
| *LS* | (-)-Limonene synthase | CGGTGGTGGAGAAATACTGGGTTT | CCGTAATCAGAGCGTGACTTTGC |
| *MFS* | (+)-Menthofuran synthase | GCCGGAACCGATACGACTTT | TTTTAGGGTACGCGGGTTTTT |
| *MR* | (-)-Menthone/(-)-menthol reductase | CGCTGTTGCTGTTGCTCACTT | GTTTTGGGATGGAATGGATGTG |
| *PR* | Pulegone reductase | ACAGCCTGAAGCAGCCTGAA | CGGCAGAACCATCTCAAGGA |
| *S-TPS* | Putative sesquiterpene synthase | CGCAAGAGACAGAAATGTGGAG | GTTGGATAGCGTCGGTGAGAAT |
| *CHI* | Chalcone-flavanone isomerase family protein homolog to AT3G55120 | GGGGTGAGAGGGATGGAGAT | GATGTCACGGCAGTCTCCTC |
| *CHI* | Chalcone-flavanone isomerase family protein homolog to AT5G05270 | GGTGCGAGTGGTGGTGATAA | TCAGCCAAACGGTCCCTTAC |
| *UBQ* | Ubiquitin | GCCTCCGAGCAGAGCAAAC | TCTGATGAAGCATTGCGTCTATCCT |
